# Molecular Evolutionary Consequences of Island Colonization

**DOI:** 10.1093/gbe/evw120

**Published:** 2016-06-30

**Authors:** Jennifer E. James, Robert Lanfear, Adam Eyre-Walker

**Affiliations:** ^1^School of Life Sciences, University of Sussex, Brighton, United Kingdom; ^2^Department of Biological Sciences, Macquarie University, Sydney, New South Wales, Australia; ^3^Division of Evolution Ecology and Genetics, Research School of Biology, the Australian National University, Canberra, Australian Capital Territory, Australia

**Keywords:** effective population size, genetic diversity, bottlenecks, polymorphism, substitution

## Abstract

Island endemics are expected to have low effective population sizes (*N_e_*), first because some may experience population bottlenecks when they are founded, and second because they have restricted ranges. Therefore, we expect island species to have reduced genetic diversity, inefficient selection, and reduced adaptive potential compared with their mainland counterparts. We used both polymorphism and substitution data to address these predictions, improving on the approach of recent studies that only used substitution data. This allowed us to directly test the assumption that island species have small values of *N_e_*. We found that island species had significantly less genetic diversity than mainland species; however, this pattern could be attributed to a subset of island species that appeared to have undergone a recent population bottleneck. When these species were excluded from the analysis, island and mainland species had similar levels of genetic diversity, despite island species occupying considerably smaller areas than their mainland counterparts. We also found no overall difference between island and mainland species in terms of the effectiveness of selection or the mutation rate. Our evidence suggests that island colonization has no lasting impact on molecular evolution. This surprising result highlights gaps in our knowledge of the relationship between census and effective population size.

## Introduction

Island species have long been considered to be under greater threat of extinction than their mainland counterparts (Johnson and Stattersfield 1990; Frankham 1997; Mckinney 1997; Purvis et al. 2000; Jones et al. 2003). Although extinction itself is caused by a number of stochastic factors, not least human activity (Pimm et al. 1988; Burgess et al. 2013), the susceptibility of island populations may also be a consequence of population genetics. Island species are likely to have experienced population bottlenecks at some point in their evolutionary history due to founder events during the initial island colonization. As only a fraction of individuals from the original population found an island population, only a fraction of the original genetic diversity of the population will be maintained, and effective population sizes (*N_e_*) will be small (Nei et al. 1975). In addition, island species are restricted to relatively small areas, which could impose long-term restrictions on census population sizes, and in turn on long-term *N_e_*. Therefore, it may be that island species are genetically vulnerable.

Low diversity and low *N_e_* could theoretically reduce the adaptive potential of a species, as standing levels of genetic variation determine the alleles that are immediately available for evolution to act upon (Hermisson and Pennings 2005; Barrett and Schluter 2007; Messer and Petrov 2013). In addition, populations founded by a small number of individuals will experience increased inbreeding. Inbreeding results in an increasingly homozygous population, and, therefore, there is a greater risk that deleterious recessive alleles will be exposed (Charlesworth B and Charlesworth D 1987), which could have significant fitness costs. There is some evidence that bottlenecked species do experience a loss of fitness: for example, Frankham et al. (1999) demonstrated that laboratory populations of *Drosophila* showed reduced evolvability (in terms of ability to tolerate increasing concentrations of an environmental pollutant) after a bottleneck; whereas Briskie and Mackintosh (2003) uncovered a link between the severity of population bottlenecks and the loss of fitness in birds.

In addition, species with low effective population sizes are expected to have inefficient selection, resulting in high levels of deleterious mutations segregating and a tendency to fix deleterious mutations. However, past studies investigating the differences in the efficiency of selection between island and mainland species have provided only limited support for this prediction. Johnson and Seger (2001) found some evidence that island species had less efficient selection, but this was for a small and taxonomically restricted dataset. Woolfit and Bromham (2005) used a much larger and more varied dataset; however, they reported a difference between island and mainland species that was only significant at the one-tailed level, whereas Wright et al. (2009) found no significant difference between island and mainland species. This may be because previous studies have focused on substitution rates as measures of the efficiency of selection, in particular the ratio of the rate of non-synonymous substitution to the rate of synonymous substitution (*ω*). The problem with considering substitution data is that a reduction in *N_e_* is expected to increase the rate at which slightly deleterious mutations are fixed, but reduce the rate at which advantageous mutations are fixed, particularly if the rate of adaptation is limited by the supply of mutations. We, therefore, cannot make a clear prediction about the effect of *N_e_* on *ω*. This issue can be addressed by using polymorphism data instead of substitution data, using the ratio of non-synonymous to synonymous polymorphisms, because advantageous mutations, subject to directional selection, are not expected to significantly contribute to polymorphism (Kimura 1984; Kryazhimskiy and Plotkin 2008; Ho et al. 2011).

It seems likely that adaptive evolution might occur for at least some island species, despite their predicted low effective population sizes, due to the fact that the species is encountering a novel habitat. Although populations with large effective population sizes may have more efficient selection, we might also expect positive selection to play a significant role after colonization events as species adapt to new environmental requirements and ecological niches. However, in making predictions regarding adaptive evolution, it is important to consider the direction of colonization. Although island species most commonly colonize an island from a nearby mainland, occasionally lineages that originated on islands re-colonize a mainland, providing an interesting contrast in terms of molecular evolution. Species colonizing the mainland from islands are likely to experience population size increases, and, therefore, increases in *N_e_*. This could result in a spate of rapid molecular evolution in the new mainland population as advantageous mutations that were previously effectively neutral become fixed (Takano-Shimizu 1999; Charlesworth and Eyre-Walker 2007).

However, predictions about the molecular evolution of island species are predicated on the crucial assumption that island species do in fact have lower *N_e_* and levels of genetic diversity than mainland species. Whether this is in fact the case is not certain, because census population size can sometimes be a poor indicator of genetic diversity (Lewontin 1974; Bazin et al. 2006; Leffler et al. 2012; Romiguier et al. 2014). Although some studies uncover a link between the two (for overview, see Frankham 2012), other authors have not found a relationship; for example, Bazin et al. (2006) and Nabholz et al. (2008) failed to find any strong relationship between mitochondrial diversity and traits associated with *N_e_* (such as body mass), or between diversity and IUCN category, an index partly based on assessments of census population size. More generally, there is surprisingly little variation in levels of diversity between species; one recent paper reported a range of nucleotide diversities of 800-fold across a range of taxa, with most species falling within a range of 50-fold, many orders of magnitude smaller than their estimated census population size differences (Leffler et al. 2012). The determinants of genetic diversity remain poorly understood.

One possible complicating factor is the mutation rate. Both Nabholz et al. (2008) and Romiguier et al. (2014) found evidence suggesting that there are lineage-specific differences in the mutation rate, in mitochondrial and nuclear data, respectively. How the mutation rate evolves is contentious: if selection is responsible for determining the mutation rate, populations with high effective population sizes should have the lowest mutation rates, because selection will be more effective at reducing the rate (Lynch 2010). This is because whether a mutation can be selected depends on the strength of selection being greater than 1/*N_e_*. However, support for this prediction remains mixed. For example, in previous studies of island–mainland systems (all of which controlled for phylogenetic non-independence), two found no difference in substitution rate between island and mainland lineages (Johnson and Seger 2001; Woolfit and Bromham 2005), whereas another found that it was mainland species that had higher rates of substitution (Wright et al. 2009), the opposite of what we might expect if the mutation rate depends on the population size. Another factor that may contribute to unexpected patterns of diversity is selection at linked sites: this reduces genetic diversity, particularly in genomic regions with low rates of recombination (Maynard Smith and Haigh 1974; Gillespie 2000; Frankham 2012). On one hand, linked selection may occur more frequently in populations with high values of *N_e_*, reducing diversity more rapidly than in populations with a low *N_e_* (Corbett-Detig et al. 2015). On the other hand, it could be that selective sweeps occur more commonly in species adapting to a new environment e.g. Montgomery et al. (2010).

In summary, we expect island species to have low effective population sizes and because of this, we expect them to have low levels of genetic diversity. We also expect selection to be less efficient in island species, leading to higher ratios of non-synonymous to synonymous polymorphism, and potentially to increases in the mutation rate (the mutation rate might increase to such an extent that island and mainland species have similar diversities, but this is expected to take some time to occur). Whether we expect island species to have higher ratios of non-synonymous to synonymous substitution depends on how much adaptive evolution there is, and how this is affected by *N_e_* and the act of colonization. If there is no adaptive evolution, then island species are expected to have higher values of *ω*; however, adaptive evolution could potentially be either reduced in island species because of their low effective population sizes, or increased because of adaptation to a new environment, given that in most cases the island is the new environment that is colonized. Here we perform the first analysis of polymorphism data from a dataset of phylogenetically independent pairs of island and mainland species, and combine this with substitution data. The paired study design is crucial: there are a large number of life history traits that are known to influence molecular evolution (e.g. body size, fecundity and generation times) and could, therefore, act as confounding factors (Bromham 2011; Lanfear et al. 2013). Closely related island and mainland species have similar life-history traits, and even if there is variation, it is not expected to be systematic, and so should not bias our results. Therefore, island colonization itself should be the primary reason for any differences in molecular evolution between island and mainland species (Johnson and Seger 2001; Woolfit and Bromham 2005).

## Methods

### Dataset

The dataset was compiled by combining all the independent island–mainland species comparisons used in two previous studies: 33 from Wright et al. (2009) and 34 from Woolfit and Bromham (2005). This dataset was then expanded using a keyword search (“endemic”) of the Arkive species database (http://www.arkive.org/, last accessed June, 2014). One or more mainland relatives and outgroup species were then identified for each island species. This added 45 species comparisons to the dataset. Some comparisons contained a single island and mainland species, whereas some consisted of multiple island and/or mainland species. All phylogenies were checked for agreement with the literature, and apparent direction of colonization was noted. In addition, the recorded range area of the species used was calculated from IUCN records (IUCN 2014) using ArcGIS (ESRI 2011). Endemic species of islands with very large areas (such as Madagascar) were excluded on the grounds that these species are unlikely to experience restricted ranges. The endemic species with the largest ranges in this analysis are found on Cuba. Protein-coding sequences were collected from NCBI (www.ncbi.nlm.nih.gov/genbank/, last accessed August, 2014). Sequences were collected if there was an orthologous gene available for each of the island, mainland and outgroup species in a comparison, or if there were multiple sequences of the same loci available for both the island and the mainland species in a comparison. A note was made of whether the sequences were nuclear, mitochondrial or chloroplast. All alignment files are available at: http://dx.doi.org/10.6084/m9.figshare.1296151.

### Statistical Tests

This study has a paired design, in that each island species/clade is compared with a closely related mainland species/clade, with each comparison occurring only once in each analysis. If a choice had to be made between comparisons (for example, if statistics from both the mitochondrial and nuclear genomes were available for a single comparison) the statistics that corresponded to the longest sequence alignment were used. This decision should reduce sampling error, because longer sequences are more representative than short sequences. We also calculated a relative value for each comparison, because values can differ considerably between different island–mainland comparisons. To do this, we divided each island statistic by the sum of the island and mainland statistics; e.g. if the statistic being considered is X (for example, the nucleotide diversity), we calculate the relative value as *X*’(island) = *X*(island)/(*X*(island) + *X*(mainland)). Using this method, if the island and mainland values are the same, then the relative island value will be 0.5. Therefore, to quantify the difference between island and mainland values, we used a Wilcoxon signed-rank tests to assess whether the distribution of relative island values was significantly different from a distribution that is symmetrical about 0.5. In order to assign confidence intervals to our results we bootstrapped the data, using 1,000 bootstrap datasets. For each bootstrap, the relative island values were randomly resampled (with replacement), and the mean of the relative values was calculated.

### Polymorphism Data

Sequences (of the same loci from the same species) were aligned by eye using Geneious; the alignment was then analyzed using our own scripts. A number of statistics were recorded, including nucleotide diversity and number of polymorphisms. If a comparison included multiple island and/or multiple mainland species, average values of each statistic were taken across the species. Similarly, if multiple sequences from the same genome were available for a particular island/mainland comparison, the average value of the sequences was used. Therefore, each comparison is represented by a single island, mainland and outgroup value of each polymorphism statistic for a particular genome.

The data was used to calculate *π_N_*/(*π_N_*+ *π_S_*), where *π_N_* is the non-synonymous diversity and *π_S_* is the synonymous diversity. This ratio is used because, unlike polymorphism counts, nucleotide diversity is unbiased by the number of chromosomes sampled. In addition, using total diversity as the denominator reduces the number of undefined values to those comparisons in which both the island and mainland species had no diversity and were, therefore, uninformative. Any comparisons with undefined values were excluded from the analysis.

### Substitution Data

Substitution data were calculated by aligning orthologs of island, mainland, and outgroup species. If multiple sequences at different loci were available for all of the species in a comparison, sequences were concatenated prior to alignment; however, sequences from different genomes of the same organism were treated separately. The alignments were pruned so that they included equal numbers of island and mainland species to control for the node-density effect (Hugall and Lee 2007), and then used to generate phylogenetic trees with RaxMl (Stamatakis 2014), in combination with PartitionFinder (Lanfear et al. 2012). The trees were subsequently used to run the codeml progamme of PAML version 4.7 (Yang 2007), which calculated *ω* (*d_N_*/*d_S_*) for island, mainland, and outgroup branches of each tree, as well as separate *d_N_* and *d_S_* values for each branch.

### Adaptive Evolution Tests

Polymorphism and substitution data were combined to test for differences in levels of adaptive evolution between island and mainland species. A variant of the direction of selection (DoS) statistic was used, calculated as the following: DoS = *d_N_*/(*d_N_*+* d_S_*) – *π_N_*/(*π_N_*+*π_S_*) (Stoletzki and Eyre-Walker 2011). This statistic has the advantage over using the neutrality index in that it is defined for all datasets in which there is at least one substitution and one polymorphism, so fewer species comparisons had to be excluded; it is also expected to be unbiased (Stoletzki and Eyre-Walker 2011). Positive values indicate that the dynamics of evolution are dominated by positive selection and negative values that slightly deleterious mutations predominate.

## Results

### Dataset Overview

To investigate the consequences of island colonization on molecular evolution, we compiled data for 112 island–mainland comparisons. In approximately 90% of cases, the inferred direction of colonization is from mainland-to-island. The data are dominated by mitochondrial sequences from birds, which comprise 40% of the species comparisons (table 1a), but we have a reasonable number of mitochondrial sequence comparisons available for invertebrates (11%) and (non-avian) reptiles (13%), and a moderate number of nuclear sequence comparisons (approximately 20% of all available comparisons are nuclear DNA). The sequences used in this analysis are on average 750 nucleotide bases long. For 70 of our comparisons, multiple sequences from the same species were available, allowing us to conduct polymorphism analyses. The mean number of sequences available per species was 7. Again, this dataset is dominated by mitochondrial data from birds (table 1b). For a full list of species used in this analysis, see the archived data at: http://dx.doi.org/10.6084/m9.figshare.1296151.

**Table 1:** (a) and (b) An Overview of the Sequences Gathered in This Analysis, Split by DNA Type and Taxonomic Group. For Analyses that Combined Data Across DNA Types, Each Species Comparison Appeared Only Once: The Numbers of Sequences Available in These Cases Are Given in the “Combined Dataset” Column. When Choosing Between Sequences from Different Genomes for a Particular Comparison, We Always Used the Longest Sequence

(a)				
Divergence	Mitochondrial	Nuclear	Chloroplast	Combined Dataset
Amphibian	1	2	—	2
Bird	60	9	—	60
Invertebrate	15	3	—	15
Mammal	2	2	—	2
Plant	—	2	10	12
Reptile (non-avian)	18	14	—	21
Total	96	32	10	112

(b)				
Polymorphism	Mitochondrial	Nuclear	Chloroplast	Combined Dataset
Amphibians	—	1	—	1
Bird	37	2	—	37
Invertebrate	11	1	—	11
Mammal	1	—	—	1
Plant	—	1	4	4
Reptile (non-avian)	11	9	—	16
Total	60	14	4	70

### Geography

**Fig. 1.**
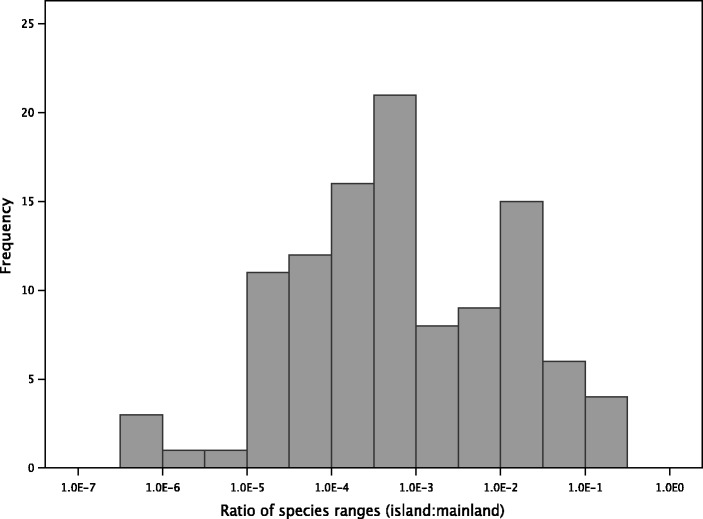
The frequency distribution of the ratios of island:mainland species range areas.

Island species are studied from a molecular evolutionary perspective because they are expected to have smaller populations than mainland species due to their small ranges. However, this assumption is rarely tested. In this study, the ranges of the species used were confirmed where possible using the IUCN database (IUCN 2014). The mean range of island species was 5,780 km^2^, whereas for mainland species, this mean range was over 4,080,000 km^2^. The ratio of island to mainland range sizes did not exceed 0.25 for any of the comparisons used, and in the majority of cases, island species had ranges which were less than 1% of the area of those of their mainland counterparts (fig. 1). Therefore, we have evidence that the island species used in this study inhabit substantially smaller geographic regions than their mainland relatives, although we have no information on population density.

### Synonymous Diversity (*π*_S_)

We might expect island species to have lower diversity than their mainland counterparts for two reasons. First, island species inhabit substantially smaller areas than their mainland relatives, resulting in a smaller census population size and hence potentially a smaller long-term *N_e_*. Second, island populations are likely to be founded by few individuals, which again is expected to result in a small *N_e_*. Because diversities can differ quite substantially between phylogenetic groups, we calculated relative values of island diversity from each comparison by dividing each by the sum of the island and mainland diversities. Therefore, if island *π*_S_ is significantly smaller than mainland *π*_S_, the relative island values will be significantly lower than 0.5.

As expected, we find that island species have significantly lower *π_S_* for both our combined dataset, and when we consider mitochondrial and nuclear DNA separately (table 2). Chloroplast sequences show the opposite pattern, but as there are only two comparisons, this is likely to be due to sampling error. When different taxonomic groups were considered separately, island birds and island reptiles both had significantly lower *π_S_* than their mainland counterparts, whereas there was no significant difference between island and mainland invertebrates (table 2) (for other groups we do not have enough data to make a valid comparison). Although we find that island species have lower diversity than mainland species, there is no significant correlation between relative island diversity and the ratio of the island and mainland ranges, either overall or for any subset of the data (see table 2). However, despite being statistically significant, the differences between mainland and island species are relatively modest. Island species have a mean *π*_S_ that is only 31% smaller than that of mainland species, and in about one-third of cases, island species have higher *π_S_* than their mainland relatives.

**Table 2:** Differences in Synonymous Nucleotide Diversity (*π_S_*) Between Island and Mainland Species. The Number of Comparisons Used in Each Analysis is Given in the Second Column (*n*). The Mean Relative Value of Island *π_S_* is Given in the Fifth Column, with Relative Values Calculated as: (island *π_S_*)/(island *π_S_*+mainland *π_S_*). Any Undefined Values were Excluded from the Analysis. CIs for the Relative Island Values of *π_S_* Are Given in the Sixth and Seventh Columns. A One- Tailed Wilcoxon Signed-Ranks Test on the Relative Island Values was Conducted, with the Alternative Hypothesis that the True Island Value is Less Than 0.5. The *P* value of This Test Is Given in the Eighth Column, with Any Statistically Significant Results Highlighted in bold. Spearman’s Coefficient of Rank Correlation Between the Ratio of Island to Mainland Species Ranges and the Relative Island *π_S_* is Given in the Last Column. None of These Correlations are Statistically Significant

Dataset	*n*	Mean Island *π_S_*	Mean Mainland *π_S_*	Mean Relative Island *π_S_*	Lower CI	Upper CI	Wilcoxon P-value	Spearman’s rho of the correlation between the ratio of ranges and relative island *π_S_*
Combined	70	0.027	0.039	0.36	0.29	0.45	**0.0013**	0.14
Chloroplast	2	0.0023	0.00058	0.85	0.69	∞	1	—
Mitochondrial	60	0.032	0.052	0.38	0.29	0.46	**0.0041**	0.15
Nuclear	14	0.0015	0.0069	0.22	0.039	0.45	**0.039**	0.039
Bird	37	0.011	0.028	0.34	0.24	0.45	**0.0035**	0.041
Invertebrate	11	0.078	0.058	0.53	0.33	0.73	0.69	0.3
Reptile (non-avian)	16	0.037	0.052	0.27	0.095	0.44	**0.018**	0.034

It is potentially possible to differentiate between the two possible causes of the lower diversity in island species by considering the ratio of island to mainland nucleotide diversity as a function of the time of divergence between the island and mainland species. In this analysis, we use the level of synonymous divergence between island and mainland species/clades (*d_S_*) as an estimator of the time at which species diverged because we lack information on colonization times. However, it should be noted that this is a crude estimator of the divergence time because *d_S_* is dependant on both the time of divergence and the mutation rate.

**Fig. 2.**
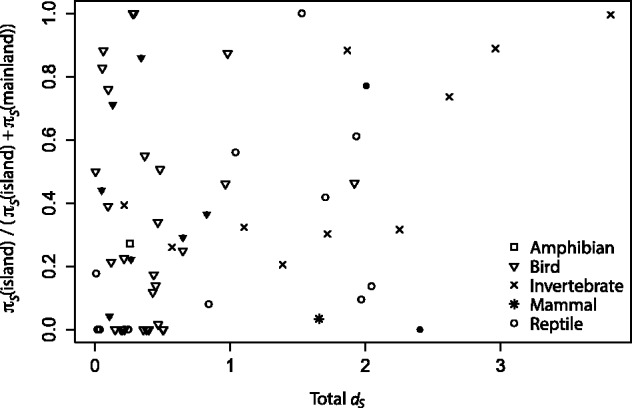
The ratio of island diversity to the combined island and mainland diversity, *π_S_*(island)/(*π_S_*(island)+ *π_S_*(mainland)), where *π_S_* is the synonymous diversity, plotted against total divergence (*d_S_*) between island and mainland species. Filled shapes indicate comparisons in which the inferred direction of colonization is island to mainland.

If most of the reduction in diversity is due to a bottleneck during colonization, then we expect the difference in island to mainland diversity to be greatest when the evolutionary divergence is shortest. In contrast, if diversity is largely determined by population sizes after colonization then we might expect the ratio of island to mainland diversity to decline with evolutionary divergence. Consistent with the bottleneck hypothesis, we find that relative island synonymous diversity, *π_S_*(island)/(*π_S_*(island)+*π_S_*(mainland)), a measure of the island diversity relative to mainland diversity, which is defined for all informative comparisons, is positively correlated to the synonymous divergence between island and mainland species across our combined dataset (Pearson’s correlation, *r*= 0.318, *P*= 0.012) (fig. 2). The correlation increases in strength if we restrict the analysis to mainland-to-island colonization events (*r* = 0.384, *P* = 0.004), and is negative, although non-significant, if we consider colonizations that occurred in the opposite direction (*r* = −0.129, *P* = 0.74). However, as there are few comparisons available in which the direction of colonization is inferred to be island to mainland, we probably lack power to detect any significant trends in this group (see fig. 2). The positive correlation that we have found appears to be driven by a group of island species/clades that are closely related to their mainland relatives, and are, therefore, likely to be recent colonists, and have no synonymous diversity (fig. 2), because the positive correlation disappears when species with no synonymous diversity are removed from the analysis (*r* = 0.214, *P* = 0.150). Although the low levels of diversity we have recorded could be a result of low levels of mutation and/or short sequences, this explanation is unlikely because we would expect equal numbers of island and mainland species to have low diversity (i.e. in fig. 2 we would expect an equal number of points clustering at 1 on the *y* axis as at 0), which is not what we observe.

Reptiles are disproportionately represented among the species with no genetic diversity in the island species/clades (6 out of 14 reptiles compared with 9 out of 35 birds and 0 out of 10 invertebrates). If each phylogenetic group is considered individually, we find a significant positive correlation between relative island diversity and *d_S_* for invertebrates (*r* = 0.752, *P* = 0.012) and positive but non-significant correlations for birds and reptiles (fig. 2) (we do not have enough data to study the other groups individually). As a group, birds appear to retain the highest levels of diversity, with some species seemingly not undergoing a population bottleneck during the colonization event, perhaps because there are more individuals initially founding the island population and/or because there is continued migration from the mainland. This is compatible with the greater dispersal ability of birds compared with other animal groups. Reptiles, on the contrary, appear to experience a quite severe loss of diversity during founder events (fig. 2).

Although our results are consistent with the idea that the genetic diversity of island species is able to recover over time, either through continued immigration or the accumulation of new genetic diversity *in situ*, an alternative interpretation is that island species that are not diverse simply go extinct. This may be why only young species have low levels of diversity (out of 62 comparisons, only the chameleon *Archaius tigris* was moderately divergent without any synonymous diversity at all). These explanations are not necessarily mutually exclusive. Nevertheless, it is surprising that aside from those species with no synonymous diversity, in most cases, island species have similar and, in some cases, more genetic diversity than their mainland counterparts. If we remove the comparisons in which island diversity is zero and re-analyze the data, we find that the remaining island species do not have lower synonymous diversity than mainland species (Wilcoxon signed rank test, *n* = 48, *P* = 0.32). This suggests that island species/clades only have lower levels of diversity if they have recently (in terms of generations) undergone a population bottleneck.

### Effective Population Sizes

The fact that the genetic diversity of island species is generally not lower than that of mainland species suggests that they do not have lower effective population sizes. To investigate this, we estimated *N_e_* by dividing *π*_S_ by *d_S_* (using *d_S_*, synonymous divergence, to approximate the mutation rate) and compared island species with their mainland counterparts. Note that these effective population size estimates can only be compared against each other (i.e. within each island-mainland comparison), because in effect, we are dividing the diversity by the product of the mutation rate per generation and the number of generations because the mainland and island species diverged. Mainland species had significantly greater effective population sizes than island species overall (Wilcoxon signed-ranks test, *n* = 66, *P* = 0.030); however, the differences are small; on an average, we estimate island species to have an effective population size that is 69% that of mainland species (95% CIs: 51%, 89%). If we exclude those comparisons in which the island species had no synonymous diversity, the difference between island species and mainland species is no longer significant (*n* = 48, *P* = 0.566).

### Efficiency of Selection

Selection is expected to be less efficient in species with small *N_e_*. However, we have found little evidence to suggest that island species have lower long-term effective population sizes than mainland species. It is, therefore, perhaps not surprising that we find little evidence for selection being less efficient in island species. Using polymorphism data, we compared *π_N_*/(*π_N_*+*π_S_*) between island and mainland species and found that island species did not have significantly larger values of *π_N_*/(*π_N_*+*π_S_*) (Wilcoxon signed-ranks test, *n* = 48, *P* = 0.54). We also found no difference when considering different DNA types separately, or when considering different taxonomic groups separately (table 3). We also find no correlation between the relative island value of *π_N_*/(*π_N_*+*π_S_*) and the ratio of island and mainland range sizes (results not shown). It should be noted, however, that most of the island species that have no synonymous polymorphisms also have no non-synonymous polymorphisms and hence are excluded from the analysis because *π_N_*/(*π_N_*+ *π_S_*) is undefined.

**Table 3:** Differences in *π_N_*/(*π_N_*+ *π_S_*) Between Island and Mainland Species. The Number of Comparisons Used in Each Analysis is Given in the Second Column (*n*). The Mean Relative Values of Island *π_N_*/(*π_N_*+ *π_S_*) is Given in the Fifth Column, with Relative Values Calculated as: (Island *π_N_*/(*π_N_*+ *π_S_*))/(Island *π_N_*/(*π_N_*+ *π_S_*) + Mainland *π_N_*/(*π_N_*+ *π_S_*)). Any Undefined Values Were Excluded from the Analysis. CIs for the Relative Island Value of *π_N_*/(*π_N_*+ *π_S_*) are Given in the Sixth and Seventh columns. A One- Tailed Wilcoxon Signed-Ranks Test on the Relative Island Values was Conducted, with the Alternative Hypothesis that the True Island Value is Greater than 0.5. Statistically Significant Results are Highlighted in Bold

Dataset	n	Mean Island *π_N_*/(*π_N_*+*π_S_*)	Mean Mainland *π_N_*/(*π_N_*+*π_S_*)	Mean relative island *π_N_*/(*π_N_*+*π_S_*)	Lower CI	Upper CI	Wilcoxon *P*-value
**Combined**	48	0.18	0.093	0.50	0.40	0.60	0.54
**Chloroplast**	1	0.26	0.22	0.54	—	—	—
**Mitochondrial**	44	0.17	0.092	0.50	0.40	0.60	0.51
**Nuclear**	3	0.18	0.13	0.39	0	∞	0.75
**Bird**	28	0.27	0.10	0.54	0.40	0.67	0.32
**Invertebrate**	10	0.035	0.055	0.54	0.33	0.73	0.36
**Reptile (non-avian)**	7	0.027	0.095	0.32	0.10	0.59	0.88

We also find no significant differences between island and mainland species for *ω* (non-synonymous divided by synonymous divergence) overall, or if we split the data by phylogenetic group or genome type (table 4). However, there is an expectation that *ω* will increase during a population size expansion (Takano-Shimizu 1999; Charlesworth and Eyre-Walker 2007) and so we might expect island-to-mainland colonizations to show different patterns to mainland-to-island colonizations. If we restrict our analysis to mainland-to-island colonizations, we still do not observe a significant difference between island and mainland *ω* overall, or for each genome, although if we split by phylogenetic group, the result for birds is close to being statistically significant (table 4). We also do not observe any significant difference in *ω*(mainland)/*ω*(island) between species that have colonized the island from the mainland, and the mainland from the island (independent samples *t*-test, *P* = 0.315), contrary to the results of Charlesworth and Eyre-Walker (2007). We find no correlation between *ω*(mainland)/*ω*(island) and the ratio of island and mainland range sizes (results not shown).

**Table 4:** (a) and (b). Differences in *ω* Between Island and Mainland Species. The Number of Comparisons Used in Each Analysis is Given in the Second Column (*n*). The Mean Relative Value of Island *ω* is Given in the Fifth Column, with Relative Values Calculated as: (Island *ω*)/(Island *ω +*Mainland *ω*). CIs for the Relative Island Values of *ω* are Given in the Sixth and Seventh Columns. A Two- Tailed Wilcoxon Signed-Ranks Test on the Relative Island Values was Conducted, to Test Whether the Distribution of Island Values was Significantly Different from Symmetrical about 0.5. Statistically Significant Results are Highlighted in Bold. In (a), the Total Dataset is analyzed and then Divided by DNA Type and Taxonomic Group, whereas in (b), the Comparisons are Split by Colonization Direction; I→M Refers to Comparisons in Which the Colonization Direction was Island-to-Mainland, Whereas M→I is Mainland-to-Island. Where the Colonization Direction was Mainland-to-Island, Comparisons were Further Divided by Genome and Taxonomic Group

Dataset	*n*	Mean Island *ω*	Mean Mainland *ω*	Mean Relative Island *ω*	Lower CI	Upper CI	Wilcoxon *P*-Value
(a)							
Combined	112	0.10	0.087	0.53	0.50	0.57	0.20
Chloroplast	10	0.34	0.16	0.70	0.57	0.83	0.11
Mitochondrial	96	0.042	0.051	0.52	0.49	0.57	0.38
Nuclear	32	0.37	0.24	0.52	0.43	0.62	0.68
Bird	60	0.083	0.062	0.54	0.50	0.59	0.17
Invertebrate	15	0.059	0.028	0.54	0.41	0.66	0.85
Plant	12	0.31	0.17	0.66	0.53	0.76	0.18
Reptile (non-avian)	21	0.092	0.11	0.50	0.41	0.59	0.76

(b)							
I→M	14	0.16	0.19	0.45	0.34	0.57	0.50
M→I	98	0.095	0.071	0.54	0.49	0.60	0.11
M→I Chloroplast	9	0.26	0.15	0.69	0.49	0.87	0.20
M→I Mitochondrial	84	0.040	0.035	0.54	0.47	0.60	0.20
M→I Nuclear	29	0.39	0.23	0.53	0.39	0.66	0.62
M→I Bird	51	0.088	0.044	0.56	0.50	0.62	0.058
M→I Invertebrate	15	0.059	0.028	0.54	0.36	0.71	0.85
M→I Plant	11	0.24	0.16	0.64	0.48	0.80	0.32
M→I Reptile (non-avian)	17	0.069	0.073	0.51	0.37	0.64	0.85

### Adaptive Evolution

Given that there seems to be little difference in *N_e_* between island and mainland species, we might expect colonization of an island to lead to a burst of adaptive evolution, because the colonizers are experiencing a new environment that might have empty niches into which the species can adaptively evolve (this effect might have been reduced or eliminated if island species had lower *N_e_* and rates of adaptation were mutation limited). To investigate whether colonization leads to higher rates of adaptive evolution, we estimated the rate of adaptive amino acid substitution along the island and mainland lineages using two approaches. First, we calculated the direction of selection (DoS) statistic for each lineage. We find that on an average, DoS is negative in both island and mainland species (table 5), indicating that slightly deleterious mutations are prevalent in our data. We find no significant difference in values of DoS between island and mainland species, either when considering the dataset as a whole, or when the results are analyzed separately depending on the direction of colonization. However, DoS is sensitive to slightly deleterious mutations segregating in the population, and, therefore, any changes in the relative frequencies of deleterious mutations between island and mainland species will influence DoS, potentially masking a signal of adaptive evolution (Nielson 2005). Unfortunately, we did not have sufficient polymorphism data to correct for slightly deleterious mutations by removing low-frequency polymorphisms (Fay et al. 2001; Charlesworth and Eyre-Walker 2008) or applying more sophisticated methods that use the site frequency spectrum to estimate the distribution of fitness effects.

**Table 5:** Differences in DoS Between Island and Mainland Species, for the Combined Dataset, and for the Dataset Split by the Direction of Colonization. The Number of Comparisons Used in Each Analysis is given in the Second Column (*n*), with the Significance Level of the Wilcoxon Signed-Ranks Test Given in the Last Column. I→M Refers to Comparisons in which the Colonization Direction was Island-to-Mainland, Whereas M→I is Mainland-to-Island

Dataset	*n*	Mean Island DoS	Mean Mainland DoS	*P* value
Combined	50	−0.090	−0.056	0.619
I → M	8	−0.053	−0.020	0.401
M → I	42	−0.106	−0.063	0.827

### Mutation Rate

We also investigated potential differences in the mutation rates of island and mainland species. In this study, we inferred the mutation rate from *d_S_*, the number of synonymous substitutions, along the lineages leading to the mainland and island species (and where there were multiple island and mainland species, from their averages). *N_e_* is predicted to influence mutation rate, and as we found no consistent differences in *N_e_* between island and mainland species we do not expect mutation rate to differ between the two groups. This is in fact the case: comparing *d_S_* values between island and mainland species revealed no significant difference (table 6, *n* = 111, *P* = 0.45). However, when different genomes were considered separately, there was one statistically significant difference between island and mainland species for nuclear DNA (*n* = 30, *P* = 0.01). The trend in this instance was for mainland species to have higher values of *d_S_* than island species.

**Table 6:** Differences in *d_S_* Between Island and Mainland Species. The Number of Comparisons Used in Each Analysis is Given in the Second Column (*n*). The Mean Relative Value of Island *d_S_* is Given in the Fifth Column, with Relative Values Calculated as: (Island *d_S_*)/(Island *d_S_+*Mainland *d_S_*). CIs for the Relative Island Values of *d_S_* are Given in the Sixth and Seventh Columns. A Two-Tailed Wilcoxon Signed-Ranks Test on the Relative Island Values was Conducted, to test Whether the Distribution of Island Values was Significantly Different from Symmetrical about 0.5. Statistically Significant Results are Highlighted in Bold

Dataset	*n*	Mean Island *d_S_*	Mean Mainland *d_S_*	Mean Relative Island *d_S_*	Lower CI	Upper CI	Wilcoxon *P* value
Combined	111	0.35	1.15	0.49	0.45	0.53	0.45
Chloroplast	10	0.016	0.013	0.56	0.37	0.76	0.72
Mitochondrial	96	0.56	1.42	0.49	0.45	0.53	0.75
Nuclear	30	0.058	0.16	0.40	0.31	0.50	**0.010**

## Discussion

It is generally assumed that island species will have smaller effective population sizes than mainland species. Island species are expected to have low effective population sizes initially because they are likely to have been founded by a small number of individuals (one pregnant female is sufficient) and hence experience a bottleneck. We find some evidence for this: some island species, which are very closely related to their mainland counterparts, have little or no diversity, consistent with these species experiencing extreme bottlenecks during colonization. However, besides these species, island species have similar levels of diversity to mainland species. There is no evidence to suggest that island species have low long-term effective populations sizes, despite the fact that island species occupy considerably smaller ranges than mainland species; in this analysis, island species had ranges of on average 0.14% of the area of their mainland counterparts. Consistent with island and mainland species having similar effective population sizes, we find no evidence that natural selection is less efficient in island species.

For most of our comparisons, we have a single gene, and hence little data. It is, therefore, important to consider whether we are likely to be able to detect differences between island and mainland species if they exist. The fact that we observe a significant difference in diversity between island and mainland species suggests that we do have the ability in this analysis (table 2). However, the lower 95% confidence interval indicates that island species have at least 41% of the diversity of mainland species, far larger than the ratio of the ranges (0.14%); furthermore the difference in mainland and island diversity seems to be due to a few young island species with no diversity; if these are excluded, island species have on average 92% of the diversity of mainland species (95% CIs: 65%, 128%). For our measures of the effectiveness of selection, the upper 95% CIs suggest that *π_N_*/(*π_N_*+ *π_S_*) could be up to 50% larger in island than mainland species, and that *ω* could be up to 33% larger. To put these numbers into context, *ω* is approximately 90% larger in primates than rodents (Eyre-Walker et al. 2002) and the ratio of non-synonymous to synonymous polymorphisms, *P_N_*/*P_S_*, varies by almost 8-fold in plants (Gossmann et al. 2010) so the differences between island and mainland species are modest. The differences are also consistent with very moderate differences in *N_e_*. For example, if we assume that all mutations are deleterious (although some can be effectively neutral) and the distribution of fitness effects is a gamma distribution, then the ratio of the *ω* values from two species with effective population sizes of *N_1_* and *N_2_* is expected to be $\omega_{1}/\omega_{2}={\left(N_{1}/N_{2}\right)}^{-\beta }$ (Welch et al. 2008), where *β* is the shape parameter of the gamma distribution. Analyses of both nuclear (Eyre-Walker et al. 2006; Keightley and Eyre-Walker 2007; Boyko et al. 2008; Gossmann et al. unpublished results) and mitochondrial data (James et al. 2016), suggest that *β* < 0.5 in most species. If we conservatively assume that *β* = 0.5, a 1.33 ratio of island to mainland *ω* translates into a ratio of *N_e_* values of 0.57. In other words, it appears that we have the power to detect quite small differences in *N_e_* between island and mainland species from the measures of the effectiveness of selection that we have used, and given the amount of data that we have—if island species had an *N_e_* below half that of their mainland relatives then we should have been able to detect it.

Our results are perhaps not surprising. It is well established that the relationship between population size and genetic diversity is not straightforward, with levels of genetic diversity remaining remarkably constant across groups of organisms which are incredibly disparate in terms of census population size (Lewontin 1974; Gillespie & Ohta 1996; Bazin et al. 2007; Leffler et al. 2012). What is unique about the current data is that only closely related species are compared with each other—many of the island and mainland species pairs are in the same genus. They are, therefore, likely to share life history traits, many of which influence molecular evolution. In addition, our paired study design allows us to correct for phylogenetic effects (Lanfear et al. 2010). This is crucial, as it has been well demonstrated that molecular evolution is influenced by taxonomy. For example, Romiguier et al. (2014) demonstrated that levels of diversity differ between families but are similar within a family. Correcting for phylogenetic effects has allowed us to study the effects of island colonization on molecular evolution across a wide range of taxa.

**Fig. 3.**
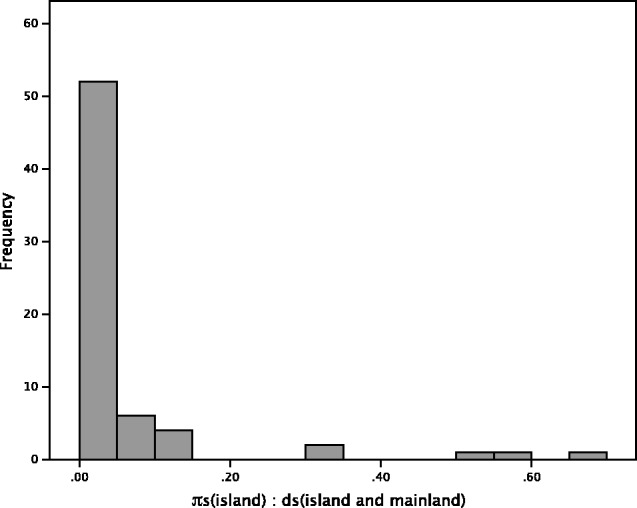
Frequency distribution of the ratio of island *π_S_*_:_ island and mainland *d_S_*.

There are a number of possible explanations for our results. It is possible that island species do not have lower effective population sizes than their mainland counterparts: if island species are commonly founded by multiple individuals, and if gene flow is maintained throughout speciation, island species might inherit much of the variation of the mainland species. We have evidence that this is true of some species: birds in particular appear to experience relatively few bottlenecks as a taxonomic group, which is probably due to their increased dispersal ability relative to other animals. However, after the initial colonization event, we might expect a reduction in the genetic diversity of island species over time, considering their restricted ranges. It is surprising that we see no evidence of this: even if we exclude those young island species with no diversity, the correlation between synonymous nucleotide diversity and synonymous divergence remains positive, but not significant (*r* = 0.214, *P* = 0.150). In addition, introgression is an unlikely explanation for the comparable neutral diversity of island and mainland species, because in the event of high levels of introgression we would expect the amount of synonymous diversity to be similar to that of synonymous divergence, assuming introgression is between the species being considered. In our analysis, the majority of species have considerably higher levels of *d_S_* than *π_S_* (fig. 3) with island *π_S_* being on average just 6% of the *d_S_* between island and mainland species. This indicates that most of the island and mainland species pairs are diverging: losing shared polymorphisms and accumulating substitutions. This pattern is not expected if there is extensive gene flow. However, it might be that there is introgression into the island from another species we have not surveyed. This is difficult to rule out.

There are also a number of factors that might obscure a relationship between effective population size and genetic diversity, which could explain our results. First, it has been suggested that levels of diversity are relatively constant across species because of an inverse relationship between population size and the mutation rate per generation (Lynch 2007; Piganeau and Eyre-Walker 2009), a relationship for which there is some evidence (Lynch 2010; Sung et al. 2012). This is hypothesized to occur because populations with large effective population sizes can more effectively select for modifiers of the mutation rate. Therefore, selection to reduce the mutation rate will be more effective in larger populations, resulting in lower mutation rates and hence levels of genetic diversity similar to those found in small populations. There is no evidence that this is the case in this analysis. When we analyzed the levels of synonymous divergence, an indicator of the neutral mutation rate, we did not find a difference between island and mainland species, indicating that island species do not have higher mutation rates. In addition, there is no evidence, from considering the efficiency of selection, that island species have lower effective population sizes. This is perhaps not surprising, because the mutation rate is expected to increase when the effective population size is reduced, but only slowly. Finally, upon excluding those species with no diversity, we do not find that diversity increases with divergence, which we might expect if higher mutation rates evolve over time in island species.

Second, it is also possible that there is selection on synonymous mutations, which might also obscure a relationship between genetic diversity and effective population size. If selection on synonymous codon use varied between sites and was directional we would find that as *N_e_* increases, the proportion of effectively neutral mutations would decrease as selection becomes more efficient. If the distribution of fitness effects of synonymous mutations was exponential, one would have a situation in which the increase in *N_e_* was perfectly matched by a decrease in the proportion of mutations that were effectively neutral (Ohta 1992). However, there is no evidence that there is selection on synonymous codon usage in animal mitochondria (Jia and Higgs 2008). Furthermore, it has been suggested that selection on synonymous codon use is stabilizing in nature, at least where the synonyms match different tRNAs (Qian et al. 2012), and under such a model we might expect the strength of selection, in terms of *N_e_s*, to remain relatively constant (Charlesworth 2013)

Finally, it is also possible that the relationship between genetic diversity and the efficiency of selection is not straightforward due to selection at linked sites (Maynard Smith and Haigh 1974; Gillespie 2000). Gillespie has argued that if the rate of adaptive evolution is mutation limited then as population sizes increase so does the rate of adaptive evolution and hence the level of genetic hitch-hiking—a phenomenon that he has termed genetic draft. Some authors have found evidence to suggest that draft has an important role in reducing genetic diversity (Bazin et al. 2006; Corbett-Detig et al. 2015). However, studies generally report that draft has relatively weak effects which may not be powerful enough to reduce genetic diversity to observed levels, particularly in nuclear DNA (Andolfatto 2007; Gossmann et al. 2011; Weissman & Barton 2012; Corbett-Detig et al. 2015); the most extensive analysis of draft in the nuclear genome has shown that draft at most reduces diversity by 73% in a survey 40 eukaryotic species (Corbett-Detig et al 2015). Furthermore, there is no evidence in our data that draft is important. First, if genetic draft was prevalent in our dataset, we might expect different patterns for the organellar genomes, which have little or no recombination, and the nuclear genome (Campos et al. 2014). However, they behave in a qualitatively similar fashion between island and mainland species (for example, see tables 2 and 3). Second, we do not find a significant difference between island and mainland species in terms of their DoS. If selective sweeps were responsible for the low diversity of mainland species, we might expect mainland species to have greater values of DoS than their island counterparts. In addition, our results indicate that it is deleterious mutations that are dominating evolutionary dynamics, rather than advantageous mutations. However, it is worth noting that the signal of adaptive evolution could be obscured by a shift in the distribution of fitness effects for island species. Correcting for this with the current dataset is difficult due to a lack of sufficient polymorphism data, although the results from our limited sample indicate that it is island species that undergo a greater degree of adaptive evolution, rather than species with large population sizes.

Romiguier et al. (2014) recently showed that geographic factors likely to influence population size are poor correlates of genetic diversity when diversity is considered across the full breadth of the animal kingdom. Surprisingly, they find that propagule size is the single best predictor of diversity. Those species with few large propagules had low genetic diversity, and those with a large number of small propagules had high genetic diversity, and were termed *K* and *r* strategists, respectively. They suggest that *K* strategists might be able to maintain smaller population sizes because they invest substantially in their offspring, whereas *r*-strategists have to maintain large population sizes on average because they are more prone to population crashes. An alternative hypothesis is that propagule size is related to population density, and that the variance in population density is far greater than the variance in population range size, so that the degree to which species differ in effective and census population sizes is largely determined by density and not range size. However, this would only explain our results if population density was on an average much higher on the islands than the mainland.

Alternatively, it may be that the mutation rate itself is an important determinant of diversity, particularly in organellar genomes (Bazin et al. 2006; Lynch et al. 2006; Nabholz et al. 2008). Although the issue is controversial, Nabholz et al. (2008) showed that the mutation rate is a major determinant of mitochondrial diversity, and as our dataset is dominated by mitochondrial sequences this could explain why we did not find a difference between island and mainland species, considering that we also did not find a difference in mutation rate between them. We found a positive correlation between the mutation rate, as measured by the rate of synonymous divergence, and levels of synonymous diversity, both for our entire dataset (*n* = 138, *r* = 0.337, *P* < 0.001), and considering mitochondrial sequences separately (*n* = 112, *r* = 0.269, *P* = 0.004), which lends some support to this theory, however, we are unable to recover this correlation if we correct for phylogenetic independence by comparing island and mainland species (i.e.*π_S_*(island)/(*π_S_*(island)+*π_S_*(mainland)) is not significantly correlated to *d_S_*(island)/(*d_S_*(island) +*d_S_*(mainland)).

In conclusion, our analysis demonstrates that island colonization has had little impact on the molecular evolution of species in this dataset. For some species, the initial colonization event results in a period of low diversity, but this effect appears to be short-lived with no discernible lasting effects. Our results confirm that census population size is a poor correlate of effective population size.
